# Nickel and aluminium mixture elicit memory impairment by activation of oxidative stress, COX-2, and diminution of AChE, BDNF and NGF levels in cerebral cortex and hippocampus of male albino rats

**DOI:** 10.1016/j.crtox.2023.100129

**Published:** 2023-10-02

**Authors:** Chidinma P. Anyachor, Chinna N. Orish, Anthonet N. Ezejiofor, Ana Cirovic, Aleksandar Cirovic, Kenneth M. Ezealisiji, Orish E. Orisakwe

**Affiliations:** a African Centre of Excellence for Public Health and Toxicological Research (ACE-PUTOR), University of Port Harcourt, PMB, 5323, Choba, Port Harcourt, Nigeria; b Department of Anatomy, Faculty of Basic Medical Sciences, College of Health Sciences, University of Port Harcourt, PMB, 5323, Choba, Port Harcourt, Nigeria; c University of Belgrade, Faculty of Medicine, Institute of Anatomy, Belgrade, Serbia; d Department of Pharmaceutical Chemistry, Faculty of Pharmacy, University of Port Harcourt, PMB, 5323, Choba, Port Harcourt, Nigeria

**Keywords:** Heavy metal mixture, Oxido-inflammatory, Acetylcholine, Neurotrophic factors, Neurotoxicity

## Abstract

This study evaluated nickel and aluminium-induced neurotoxicity, as a binary metal mixture. Twenty-eight male Sprague Dawley albino rats were weight-matched and divided into four groups. Group 1 (control) received deionized water. Group 2 and 3 received Aluminium (1 mg/kg) and Nickel (0.2 mg/kg) respectively, while Group 4 received Ni and Al mixture HMM three times a week orally for 90 days. Barnes maze tests was performed. Rats were sacrificed under pentobarbital anaesthesia, cerebral cortex and hippocampus were separated, and metal levels were measured using Atomic Absorption Spectroscopy (AAS). Malondialdehyde (MDA), catalase (CAT), glutathione content (GSH), superoxide dismutase (SOD), glutathione peroxidase (GPx), Brain Derived Neurotrophic Factor (BDNF), Nerve growth factor NGF, *cyclo*-oxygenase COX-2 and Acetylcholinesterase (AChE) were assayed using ELISA kits. Ni/Al binary mixture exposed rats showed a shorter latency period (though not significant) of 3.21 ± 1.40 s in comparison to 3.77 ± 1.11 (Ni only) and 3.99 ± 1.16(Al only). Ni/Al mixture gp had the lowest levels of Mg in both the hippocampus and frontal cortex when compared with the individual metals. In the hippocampus Al only exposed rats significantly showed p < 0.05 higher iron and Ca levels in comparison to Ni/Al mixture. Al alone significantly showed p < 0.05 lower levels of Fe but higher Ca than the Ni/Al mixture group. Exposure to Al only showed lower levels of BDNF in comparison to Ni/Al combination, whereas Ni/Al mixture gp had lower levels of NGF in comparison to the individual metals in the hippocampus. In the frontal cortex Ni only, group showed significantly lower BDNF in comparison to Ni/Al mixture whereas the mixture showed significantly lower NGF when compared with Al only group. There were higher levels of COX-2 in the Ni/Al mixture than individual metal treated rats in both hippocampus and frontal cortex. AChE levels in the Ni/Al mixture group was higher than Ni or Al only gps in the hippocampus whereas in the frontal cortex, Ni/Al exposed rats showed significantly lower AChE levels in comparison to Al only group. Ni, Al and Ni/Al mixture exhibited memory impairment by activation of oxidative stress, COX-2, and diminution of AChE, BDNF and NGF levels in cerebral cortex and hippocampus. The BDNF-COX-2 AChE signalling pathway may be involved in the neurotoxicity of Ni and Al.

## Highlights

• Ni/Al mixture had lowest levels of Mg in both the hippocampus and frontal cortex in comparison to Ni or Al only.
• Ni/Al caused significantly lower AChE levels in comparison to Al only.
• Al only showed lower levels of BDNF in comparison to Ni/Al mixture,
• Ni/Al mixture had lower levels of NGF in comparison to Ni or Al only in the hippocampus.
• The BDNF-COX-2 AChE signalling pathway may be involved in the neurotoxicity of Ni and Al.

## Graphical abstract

**Figure:**
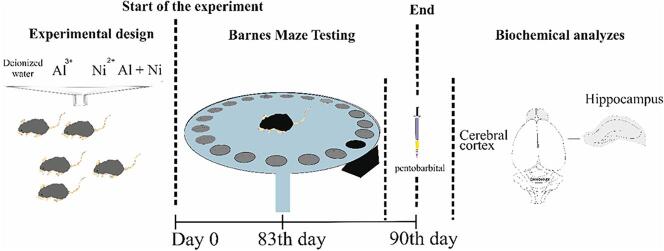

## Introduction

Aluminium (Al^3+^) is present in significant quantities in the earth's crust. Moreover, the environment is heavily polluted with Al, as it can be found in drinking water and foodstuffs, sometimes in considerable concentrations (Campos et al., 2022, Wang et al., 2020). Al exerts neurotoxic effects by inducing oxidative damage, reactive oxygen species (ROS), and neuronal death (Willhite et al., 2014) and contributes to the development of neurodegenerative and neurological diseases, including Amyotrophic lateral sclerosis (ALS), Parkinson's disease (Bittencourt and Damasceno-Silva 2022), Alzheimer’s disease (AD) (Nie 2023),dialysis dementia syndrome (DDS), autism spectrum disorder (ASD), multiple sclerosis (MS), and epilepsy.

Nickel (Ni) is a widespread environmental pollutant (Song et al., 2017, Stannard et al., 2017) commonly produced during anthropogenic processes like mining, extraction, refining, as well as being used in industrial processes such as alloy production, manufacturing of stainless steel, welding, and electroplating activities. There are concerns about the effects of Ni exposure on human health due to increased industrial application (Gates et al., 2023). The World Health Organization (WHO) reported the maximum permissible concentration of Ni in drinking water to be 0.07 mg/L. However, Ni concentrations ranging from 100 to 1000 μg/L have been recorded in mining areas, including water boiled with Ni-plated electric kettles (Berg et al., 2000, World Health Organization, 2021). The primary site of Ni toxicity is the CNS (Genchi et al., 2020). In recent years, the neurotoxicity and human carcinogenic potency of Ni and its compounds have attracted major concerns (Song et al., 2017). Earlier studies have shown the fundamental toxicity of Ni to include the initiation of oxidative stress, apoptosis, and dysfunction of the mitochondria (Ijomone et al., 2018, Xu et al., 2015).

Brain-derived neurotrophic factor BDNF a neurotrophin, synthesized and widely distributed in the brain (Martinowich and Lu 2008) plays an important role in neuronal development, neuroprotection and functional plasticity (Jin et al., 2019, Kowiański et al., 2018) and the modulation of synaptic interactions and neuroimmune axis regulation (Kowiański et al., 2018). BDNF and Nerve growth factor (NGF) might be appropriate prognostic and diagnostic markers of neurodegenerative diseases (Haridy et al., 2023). Acetylcholinesterase (AChE) is a catabolizing enzyme that promotes acetylcholine breakdown. AChE could be helpful as an early biomarker of differential diagnosis for the follow-up of patients during their early stages of cognitive impairment (Nelson et al., 2023).

It is known that redox imbalance is involved in metal-catalysed reactions in various neuropathologies There is speculation that diverse environmental factors are associated with age-related oxido-inflammatory damage to neurons. Reactive oxygen species (ROS) react with cellular macromolecules (proteins, lipids, and DNA) (Calabrese et al., 2000, Cardozo-Pelaez et al., 2000) to trigger mitochondrial damage (Lin and Beal, 2006). Heavy metals increase ROS generation and inflammation, which exacerbates the condition and constitutes a predisposing factor for neurodegenerative diseases. The exposure to chemical mixtures is a common and important determinant of toxicity and raises concerns about their introduction through inhalation and ingestion. In the environment, co-exposure is the major form of interaction between organisms and environmental toxicants (Wu et al., 2016). Therefore, joint toxicity is more practical to study than a single toxicology study.

According to the Agency for Toxic Substances and Disease Registry co-exposure to two metals at a time can have an additive (1 + 1 = 2), synergistic (1 + 1 > 2), or antagonistic (1 + 1 < 2) effect on there deleterious effects in different organs (ATSDR 2006; ATSDR2004). It is plausible to reason that simultaneous exposure to several heavy metals is more harmful than concentrating on one metal at a time. In view of the uncertainties inherent in the risk assessment process, another school of thought has however posited that at low doses, interaction between chemicals (synergy or antagonism) may be negligible. One study has shown, the range of Al and Ni concentrations to be 3100.0 to 84800.0 and 109.0 to 29749.0 ng/g adipose tissue respectively (Echeverría et al., 2021). In the GraMo cohort with highly heterogeneous distribution of metal(loid)s, increased Al and Ni concentrations were found in people living in Metropolitan Area compared to other sub-areas (Echeverría et al., 2021). Industrial and traffic-related emissions were suggested as possible sources of Al and Ni in the GraMo cohort. Al and Ni are commonly utilized/manipulated in and/or released by industrial facilities (Bencko and Yan Li Foong, 2017, Casares et al., 2005). Contamination caused by traffic is essential when studying the exposure to Al and Ni, due to the high levels of air pollution in many cities (ASCENSO). Tire wear, asphalt, road surface abrasion and braking systems are associated with significant amounts of Al, Ni among others, in air and dust contamination near roads (Hjortenkrans et al., 2007). Niger Delta, Nigeria where the present study is based, notable for severe industrial pollution arising from crude oil exploration, artisanal refining and other manufacturing outfits has a lot of similarities in terms of environmental pollution with Gramo cohort. Cooking utensils and aluminum foils used as food wrappers in Nigeria have been found to contain high Al and Ni levels (Dabonne et al., 2010, Duru and Duru, 2020). It is feared therefore that Al and Ni co-exposure may be of public health concern. This informed the choice of these two elements.

Since previous studies have demonstrated that heavy metals reach the neuronal milieu, it is essential to understand whether a mixture of Ni and Al influences the redox status in the brain, thereby hampering the cellular micro-environment and neurotrophic factors. This study has therefore evaluated the influence of Ni and Al mixture on BDNF, NGF, and AChE and the oxido-inflammatory damage of the hippocampus and frontal cortex of male albino rats.

## Materials and methods

### Chemicals

Aluminum chloride and nickel chloride (99.9 and 99.5 percent purity) were purchased from Sigma Chemical Co. (St. Louis, MO, USA). The rat ELISA kit for cyclooxygenase-2 (COX-2), acetylcholinesterase, neuronal growth factors (NGF), and brain-derived growth factors (BDNF) was purchased from Elab Science Biotechnology Company (Beijing, China). The choice of 1 mg/kg AlCl_3_·6H_2_O is premised on previous studies that demonstrated anxiogenic, depressive effects in addition to impairment of memory and spatial learning performance effects of Al begins at 0.25 mg/kg and reaches maximum at 1 mg/kg (Zghari et al 2018). Pilot studies informed the dose spacing of three times a week. This was the dose spacing that achieved appreciable bioaccumulation that was considered not excessive within the tolerable dose (JECFA 2006).

### Animals and treatments

Twenty-eight Sprague Dawley male (to avoid sex bias) albino rats aged between 7 and 9 weeks were purchased from the Department of Pharmacology, Animal House, University of Port Harcourt, Rivers State, Nigeria. The animals were housed in standard plastic cages with corn cob beddings that do not release endocrine disruptors, under room temperature of 26 ± 2 °C with a 12-hour light/dark cycle throughout the duration of the experiment, as required for adequate healthy living. The animals were acclimatized for two weeks before the commencement of the experimental procedure. A standardized protocol using the ARRIVE guidelines (Animal Research: Reporting In Vivo Experiments) checklist (Du Sert et al., 2020) was employed, and ethical approval was obtained from the University of Port Harcourt institutional Centre for Research Management and Development Animal Care and Use Research Ethics Committee with reference number UPH/CEREMAD/REC/MM86/027. The animals were administered standard feed and deionized water ad libitum.

### Experimental design

The experimental animals were weight matched and randomly divided into four groups of seven male albino rats each. Treatments were administered by oral gavage (not more than I ml at each time) to precisely administer a fixed volume of the heavy metals three times a week. The doses of the metal mixture were as follows Al (1 mg/kg) (Zghari et al., 2018) and Ni (0.2 mg/kg) (Mehrabadi). The 0.2 mg/kg Ni^2+^ used in the present study is lower than the 7–52 mg of nickel per kg of body weight per day) from 90 day-study by Velazquez and Poirer, 1994, ATSDR, 1996, 5–125 mg/kg bw/day ((Ambrose et al., 1976). and 1.3–31.6 mg of nickel per kg of body weight per day) in drinking-water (Smith et al., 1993). The 0.2 mg/kg Ni2 + is also lower than 0.25 mg/kg of Ni used by Lamtai et al., 2018, and Beidokhti and Mehrabadi, 2022 in neurobehavioral evaluation of nickel in rats.

Records of the rat weight were taken weekly, including daily feed and fluid intakes. The animals' treatment protocol detailed as follows lasted for 90 days.

### Barnes maze testing

The Barnes maze test was used to assess spatial learning and memory, as previously described in our lab (Kennard and Woodruff-Pak, 2011). The behavioral test apparatus consisted of a 91-cm-diameter gray circular plate, elevated 90 cm above the ground, with twelve identical holes (3 cm diameter), evenly spaced across the circumference of the maze. One small, dark, recessed chamber, tagged as the escape box, was located under one of the 12 identical holes and served as the goal box. The maze walls also comprised visual cues of different shapes and colors. The principle of the Barnes Maze Behavioral Testing is because the rats will employ the visual cues as they learn to locate and enter the goal box to escape the aversive environment (bright light). Rats from different groups, as stated above, were tested over 5 consecutive days, with 4 trials per day. At the beginning of each trial, the rats were placed inside a dark gray start cylinder that was lifted after about 30 s to start the trial. If the animals did not enter the correct hole within 300 s, they were gently forced to enter it and return to their home cage. During the probe trial, all holes were closed. After 300 s, the formerly correct hole was opened to let the animals return to their home cage. After each trial, the platform was thoroughly cleaned with 70 % ethanol. Decreased latencies to enter the goal box over consecutive days of testing was used as index of spatial learning and memory**.** In all the behavioral assessments observers were blinded to the study groups.

### Sample collection and brain tissue preparation

After 90 days of treatment, five rats per group were euthanized under pentobarbital anesthesia (intraperitoneal 50 mg/kg). The brain of each rat was harvested, rinsed in cold saline water, and weighed. The hippocampus and frontal cortex regions were dissected on an ice-cooled board, weighed, and divided in to two portions. One portion was used for both biochemical parameters and the other used metal analyses. The brain parts were separately homogenized in 9 vol of cold phosphate buffer (0.1 M, pH 7.4) using a homogenizer. The tissue homogenates were centrifuged at 3000 rpm for 20 min at 4 °C to separate the nuclear debris. The frontal cortex and hippocampus lysates were used for the assay of MDA, GSH, GPx, CAT, SOD, and for ELISA assays (COX-2, AChE, BDNF, and NGF) according to manufacturer’s instructions.

The Enzyme Linked Immunosorbent Assay (ELISA) kit (ELISA-Bioassay Technology Laboratory 14,780 Memorial Drive Suite 216, Houston, Texas 77079, United States) was used to measure the activity of amyloid-βeta-42 (Aβ42) (Cat.No.:E-EL-R0355 Aβ-42), Nerve growth factor (NGF) (Cat.No.:E-EL-R0355 NGF), Acetylcholinesterase (AChE) (Cat.No.:E-EL-R0355 AChE), COX-2 (cyclooxygenase-2) (Cat.No.:E- EL-R0792- COX-2), and Brain Derived Neurotrophic Factor (BDNF) (Cat.No.:E-EL-R0355 BDNF). Briefly, 100 µl of homogenized sample was added to the well and incubated for 90 min at 37 °C, the liquid was decanted after 90 min, 100 µl of biotinylated detection Ab/Ag was added and incubated for 1 h at 37 °C, the well was aspirated and washed three times, and 100 µl of Horseradish Peroxidase (HRP) conjugated was added and incubated for 30 min at 37 °C, the well was aspirated and 90 µl of substrate reagent was added and incubated at 37 °C for 15 min. The absorbance was read at optical Density (OD) 450 nm using a micro-plate reader set and the results were calculated after 50 µl of stop solution was applied.

**AChE Estimation:** This test is an improved version of the Ellman et al., (1961) method, in which AChE produces thiocholine, which interacts with 5, 5′- dithiobis (2-nitrobenzoic acid) to create a colorimetric (412 nm) product proportionate to AChE activity. Tissues were homogenized in 0.1 M phosphate buffer (PH 7.5), then centrifuged for 5 min at 5,000 rpm. The experiment was performed with clear supernatant. The absorbance was read after 1 ml of distilled water and 1 ml of calibrator were added into the cuvette, 50 µl of sample was transferred into the cuvette, and 800 µl buffer was added to the sample and mixed. Only 100 µl of chromogen was added to the samples, mixed, and incubated for 2 min before reading the first absorbance at 412 nm using a micro plate reader (EON, BIOTEK, USA). Next, 50 µl of substrate was added to the samples and left to stand for 8 min.

### Determination of body and organ weight

Animals in each group were recorded at the beginning and end of the exposure (90) using a weighing balance. The body weight of rats was measured weekly and at the end of the experiment on the 90th day, while the brain weight was recorded directly after the sacrifice on the 90th day**.**

$$Relative\;weight\;\left(\%\right)\;=\;\frac{absolute\;weight\;of\;whole\;brain\;\;(g)}{body\;weight\;(g)}\times 100$$

### Metal analyses

Thereafter, acid digestion of the brain part was carried out using 6 ml of nitric acid and 2 ml of perchloric acid (in a ratio of 3:1, respectively). After acidification, the samples were placed for 30 min before being heated at 105 °C until digestion was completed. The solution was then filtered with a Bucher funnel into a beaker using Whatman filter paper to obtain a clear solution. The solution was later made up to a final volume of 15 ml with distilled water. A Solar thermo elemental flame Atomic Absorption Spectrometer (Model SG 71906) was used to determine the levels of Al and Ni in the frontal cortex and hippocampus of the brain (Okoye et al., 2021).The optical density (OD) was measured spectrophotometrically at a wavelength of 450 nm ± 2 nm. The limits of detection (LoD) were 0.001 mg/kg for Al and Ni, while the limits of quantification (LoQ) were 0.0033 mg/kg for Al and Ni. The LOD and LOQ of calcium, iron, and magnesium were 0.001 and 0.003, respectively. Different concentrations (0.5, 1.0, 2.0, 5.0, and 10.0 mg/L) of trace elements were used for calibration of standard graphs. Absorbance values were taken at 248.3, 422.7, and 285.2 nm for iron, calcium, and magnesium, respectively, in the atomic absorption spectrometer. To verify the assay accuracy and to maintain quality, the standard solutions which were also prepared in the same acid solution as the samples were run for every 10 test samples.

### Lipid peroxidation and antioxidant analyses

The determination of oxidative stress is described briefly as follows. Lipid peroxidation in the frontal cortex and hippocampus, a well-known indicator of cellular damage from oxidative stress, was measured using thiobarbituric acid reactive substances (TBARS) as described by (Ohkawa, 1979) with some modifications by (Jamall and Smith, 1985). The method is based on the formation of a red chromophore, which absorbs at 532 nm, following the reaction of thiobarbituric acid (TBA) with malondialdehyde (MDA) and other breakdown products of peroxidised lipids. The procedure was performed as follows: 0.2 ml of frontal cortex and hippocampus homogenates were added to 0.2 ml of 8.1 % SDS, 1.5 ml of 20 % acetic acid solution (pH 3.5), and 1.5 ml of 0.8 % aqueous solution of TBA. The final volume was made up to 4.0 ml with distilled water and heated in a water bath at 95 °C for 60 min. After cooling to room temperature, one ml of sample was transferred to a tube to which an equal volume of 10 % (w/v) TBA was added (n-butanol and pyridine were added to the method from (Ohkawa, 1979). Then, the mixture was mixed again and centrifuged at 1000 g for 5 min. An aliquot of the supernatant fraction was read in a spectrophotometer (Bio-Rad, USA) at 532 nm. The lipid peroxide levels in homogenates of the frontal cortex and hippocampus samples were expressed as nanomoles of MDA per milligram of protein.

**Catalase (CAT)** activity was estimated by monitoring the rate of H_2_O_2_ breakdown at 240 nm according to Aebi's method (Bergmeyer and Bernt, 1974), with slight modifications. Briefly, 990 μg of catalase buffer (0.036 % H_2_O_2_ prepared in 50 mM phosphate buffer, pH 7.0) was added to 10 μg of frontal cortex and hippocampus lysates separately in a cuvette. Catalase activity was assayed immediately at 240 nm for 3 min. Catalase (CAT) activity was assayed by following the decrease in absorbance at 240 nm due to H_2_O_2_ consumption according to (Aebi 1983). The activity level was expressed in units per milligram protein (U/mg protein), with one unit being micromoles H_2_O_2_ consumed per minute.

The supernatant of the frontal cortex and hippocampus were used to measure glutathione peroxidase (GPx) activity by assaying glutathione recycling enzymes using cumene hydroperoxide as a substrate and monitoring NADPH oxidation at 340 nm(Ahmad and Pardini 1988). Enzyme activity was expressed as nanomoles NADPH oxidized (defined as one unit) per milligram protein. The amount of protein in the samples was determined according to Bradford 1976 (Bradford 1976).

**Reduced glutathione (GSH)** levels were estimated using Ellman's reagent 5–5-dithio-bis-2-nitrobenzoic acid (DTNB) as a coloring reagent (Moron et al., 1979). Briefly, the homogenate was precipitated with 25 % trichloroacetic acid (TCA) and centrifuged. The supernatant was taken for GSH estimation using freshly prepared DTNB solution. The intensity of the yellow color formed was read at 412 nm with a spectrophotometer. GSH concentration was expressed as nmol/mg protein using the GSH standard calibration curve.

**Superoxide dismutase (SOD)** activity was estimated with the technique previously illustrated by Misra and Fridovich (1972) (Misra and Fridovich 1972). This technique is based on the principle that at pH 10.2, SOD has the capacity to inhibit the autoxidation of epinephrine.

### Statistical analysis

All results were expressed as Mean ± Standard deviation (std). Normality test was performed prior to analyzing by ANOVA and post hoc Tukey's test. SPSS 2014 was used to perform Analysis of Variance and Tukey multiple comparison pairwise tests to check if the concentration of the biomarkers was significantly different (at a 5 % significant level) between groups. Pandas (software library written for the Python programming language for data manipulation and analysis). was used to obtain the descriptive statistical parameters (biomarkers and heavy metals mean concentration). The data analysis involved performing descriptive statistics (mean and std) on the heavy metals and biomarkers concentration before ANOVA was used to establish if there was a significant difference in the concentration of the heavy metals and biomarkers among groups.

## Results

### Effect of Ni, Al, Ni and Al mixture on the body weight, absolute and relative weight of brain, feed, and fluid intake of male albino rats

**Fig. 1:**
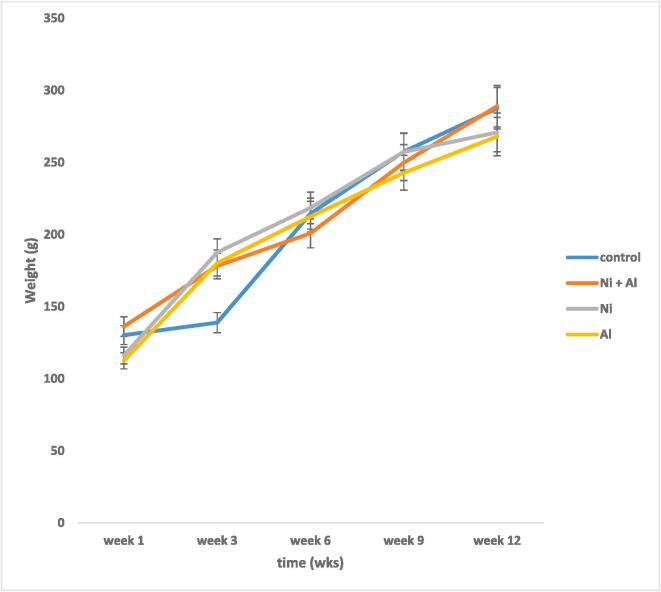
Effect of Ni + Al, Ni and Al on body weight of male Sprague Dawley rats.

Table 1 shows the effect of Ni, Al and Ni/Al binary mixture on the body weight, whole brain weight, Fluid and feed intake of rats whereas Fig. 1 shows the effect on body weight. The changes in body weight were not significant. There were also no significant changes in the fluid intake but there were significant changes in feed intake between the Ni only group and Ni/Al binary mixture group. There was a decrease in fluid and feed intakes on day 60 (not shown on Table) in comparison to day 30. But there was an increase in fluid and feed intakes on day 90 when compared with day 60. Percent body weight gain decreased in the Ni, Al and Ni, and Al HMM groups probably due to both reductions in fluid and feed intakes (Table 2).

**Table 1:** Effect of Ni, Al and Ni/Al mixture on the total brain weight (absolute and relative, Fluid and feed intake of rats exposed to Heavy Metal Mixture HMM.

Groups	Absolute Brain Weight (g)	Relative Organ Weight (%)	Feed Intake (g)	Fluid Intake (g)	
Deionized H_2_O (control)	1.84 ± 0.07^a^	1.84	220.67 ± 12.8^a^	166.98 ± 33.5^a^	
0.2 mg/kg Ni + 1 mg/kg Al	1.88 ± 0.01^a^	1.88	223.38 ± 34.3^a^	162.07 ± 37.3^a^	
0.2 mg/kg Ni	1.84 ± 0.05^a^	1.84	218.24 ± 22.6^b^	163.39 ± 22.8^a^	
1 mg/kg Al	1.87 ± 0.03^a^	1.87	219.23 ± 16.9^a^	162.49 ± 41.2^a^	
					160.01 ± 22.8^a^
					170.10 ± 43.2^a^
					160.08 ± 19.9^a^
					162.05 ± 11.7^a^
					172.11 ± 16.6^a^
					160.15 ± 15.9^a^
					168.09 ± 27.8^a^
					159.77 ± 33.5^b^
					170.75 ± 16.9^a^

Values are presented as Mean ± SD values with different superscripts are significantly different from each.

other at p < 0.05), while values with the same superscripts are not significantly different = 5.

**Table 2:** The bioaccumulation of Ni and Al (mg/kg) in the Hippocampus of male albino rats exposed to Ni, Al, and Ni & Al mixture.

Treatment	Ni (mg/kg)	Al (mg/kg)
Deionized H_2_O (control)	0.006 ±-0.002^a^	0.005 ± 0.002^a^
0.2 mg/kg Ni + 1 mg/kg Al	0.48 ± 0.14^b^	0.19 ± 0.02^b^
0.2 mg/kg Ni	0.21 ± 0.16^c^	–
1 mg/kg Al	–	0.14 ± 0.09^b^

Values = Mean ± SD, N = 5., data with different superscripts (a, b, c) are significantly different from each other (*p* < 0.05), data with the same superscripts are not significantly different; HMM = Heavy Metal Mixture.

### Barnes test

**Fig. 2:**
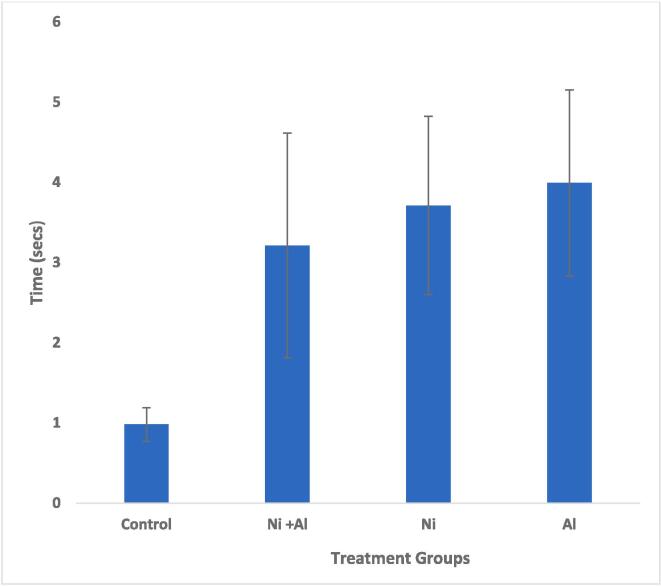
Effect of Ni, Al, and Ni & Al mixture on Barnes performance test in male albino rats.

Treatment with Ni, Al, and Ni & Al mixture showed significantly (p < 0.05) longer time in locating the escape hole indicative of impairment in learning and spatial memory in comparison to the control Fig. 2.

Table 3, Table 4 depict the bioaccumulation of Ni and Al (mg/kg) in the Hippocampus and frontal cortex of male albino rats exposed to Ni, Al, and Ni & Al mixture, respectively. There were significant (p > 0.05) increases in the levels of Ni and Al in the Hippocampus and frontal cortex after exposure to Ni, Al, and Ni & Al mixture when compared with the control group that received deionized water (Table 5).

**Table 3:** The bioaccumulation of Ni and Al (mg/kg) in the Frontal Cortex of male albino rats exposed to Ni, Al, and Ni & Al mixture.

	Ni (mg /kg)	Al (mg /kg)
Deionized H_2_O (control)	0.005 ± 0.002^a^	0.008 ±-0.001^a^
0.2 mg/kg nickel + 1 mg/kg Al (HMM)	3.40 ± 0.09^b^	1.13 ± 0.07^b^
0.2 mg/kg Ni	0.26 ± 0040^b^	–
1 mg/kg Al	–	1.28 ± 0.03^b^

Values = Mean ± SD, N = 5., data with different superscripts (a, b, c) are significantly different from each other (*p* < 0.05), data with the same superscripts are not significantly different; HMM = Heavy Metal Mixture.

**Table 4:** Iron, magnesium & calcium (mg/kg) concentrations in the Hippocampus of male albino rats exposed to Ni, Al, and Ni & Al mixture.

	Iron Conc. (mg/kg)	Calcium Conc. (mg/kg)	Magnesium Conc. (mg/kg)
Deionized H_2_O (control)	38.77 ± 35.46^a^a	133.29 ± 12.57^a^	19.77 ± 4.83^a^
0.2 mg/kg nickel + 1 mg/kg Al (HMM)	42.99 ± 9.67^b^	815.71 ± 80.99^b^	3.17 ± -1.74^b^
0.2 mg/kg Ni	43.80 ± 10.42^b^	519.25 ± 50.49^c^	12.35 ± 4.41^b^
1 mg/kg Al	95.61 ± 12.30^b^	849.90 ± 83.18^c^	16.01 ± 1.07^c^

Values = Mean ± SD, N = 5., data with different superscripts (a, b, c) are significantly different from each other (*p* < 0.05), data with the same superscripts are not significantly different; HMM = Heavy Metal Mixture.

**Table 5:** Iron, magnesium & calcium (mg/kg) concentrations in the frontal Cortex of male albino rats exposed to Ni, Al, and Ni & Al mixture.

	Iron Conc. (mg/kg)	Calcium Conc. (mg /kg)	Magnesium Conc. (mg /kg)
Deionized H_2_O (control)	178.485 ± 17.41^a^	296.16 ± 19.44^a^	37.30 ± 3.87^b^
0.2 mg/kg Ni + 1 mg/kg Al (HMM)	430.40 ± 9.36^b^	313.16 ± 21.43^b^	7.96 ± 1.64^a^
0.2 mg/kg Ni	493.35 ± 42.31^b^	416.00 ± 17.27^b^	35.05 ± 33.73^b^
1 mg/kg Al	194.81 ± 13.76^a^	410.94 ± 12.22^b^	9.64 ± 1.33^a^

Values = Mean ± SD, N = 5., data with different superscripts (a, b, c) are significantly different from each other (*p* < 0.05), data with the same superscripts are not significantly different; HMM = Heavy Metal Mixture.

Table 4 shows iron, magnesium and calcium concentrations in the Hippocampus of male albino rats exposed to Ni, Al, and Ni & Al mixture, and Magnesium concentrations in the hippocampus of male albino rats exposed to Ni, Al, and Ni & Al mixture respectively. Table 4 shows iron, magnesium and calcium concentrations in the frontal Cortex of male albino rats exposed to Ni, Al, and Ni & Al mixture, and magnesium concentrations in the frontal Cortex of male albino rats exposed to Ni, Al, and Ni & Al mixture.

Exposure to Ni, Al, and Ni & Al mixture caused significant increases in the concentrations of iron and calcium in the hippocampus and frontal cortex when compared with the control that received deionized water. There was a significant decrease in the magnesium concentrations in the hippocampus and frontal cortex following exposure to Ni, Al, and Ni & Al mixture in comparison to the control.

The effect of Ni, Al, and Ni & Al mixture on MDA and CAT, SOD, GSH, and GPx in the hippocampus and frontal cortex of male albino rats is shown on Table 6, Table 7, respectively. There were significant increases in MDA in the hippocampus and frontal cortex in the Ni, Al, and Ni & Al mixture-treated groups in comparison to the control group. Conversely, there were significant decreases in the CAT, SOD, GSH, and GPx activities in the Ni, Al, and Ni & Al mixture-treated groups in comparison to the control group.

**Table 6:** Effect of Ni, Al, and Ni & Al mixture on MDA and CAT, SOD, GSH and GPx (µmol/ml) in Hippocampus of male albino rats*.*

	MDA	CAT	GSH	GPx	SOD
Deionized H_2_O (control)	0.19 ± 0.05^a^	1.20 ± 0.970^a^	1.70 ± 0.42^a^	0.07 ± 0.03^a^	0.48 ± 016^a^
0.2 mg/kg nickel + 1 mg/kg Al (HMM)	0.57 ± 0.05^b^	0.31 ± 0.15^b^	0.50 ± 0.18^b^	0.01 ± 0.003^b^	0.15 ± 0.02^b^
0.2 mg/kg Ni	0.51 ± 0.04^b^	0.29 ± 0.082^b^	0.39 ± 0.11^C^	0.02 ± 0.001^b^	0.11 ± 0.003^b^
1 mg/kg Al	0.52 ± 0.02^b^	0.16 ± 0.04^b^	0.33 ± 0.03^c^	0.02 ± 0.00^b^	0.16 ± 0.07^b^

Values = Mean ± SD, N = 5., data with different superscripts (a, b, c) are significantly different from each other (*p* < 0.05), data with the same superscripts are not significantly different; HMM = Heavy Metal Mixture.

**Table 7:** Effect of Ni, Al, and Ni & Al mixture on MDA and CAT, SOD, GSH and GPx (µmol/ml) in Frontal Cortex of male albino rats.

	MDA	CAT	GSH	GPx	SOD
Deionized H_2_O (control)	0.25 ± 0.17^a^	0.88 ± 0.17^a^	1.78 ± 105^a^	0.07 ± 0.03^a^	0.46 ± 0.04^a^
0.2 mg/kg Ni + 1 mg/kg Al	0.56 ± 0.23^b^	0.39 ± 0.12^b^	0.38 ± 0.02^b^	0.02 ± 0.005^b^	0.18 ± 0.03^b^
0.2 mg/kg Ni	0.56 ± 0.24^b^	0.19 ± 0.01^b^	0.40 ± 0.1^b^	0.03 ± 0.014^b^	0.18 ± 0.01^b^
1 mg/kg Al	0.47 ± 0.19^b^	0.37 ± 0.17^b^	0.48 ± 0.08^b^	0.03 ± 0.15^b^	0.17 ± 0.01^b^

Values = Mean ± SD, N = 5., data with different superscripts (a, b, c) are significantly different from each other (*p* < 0.05), data with the same superscripts are not significantly different; HMM = Heavy Metal Mixture.

**Fig. 3:**
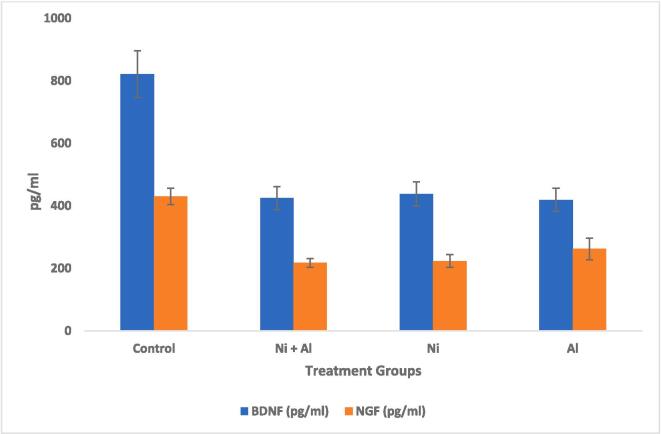
Effect of Ni, Al, and Ni & Al mixture on BDNF & NGF in Hippocampus in male albino rats.

**Fig. 4:**
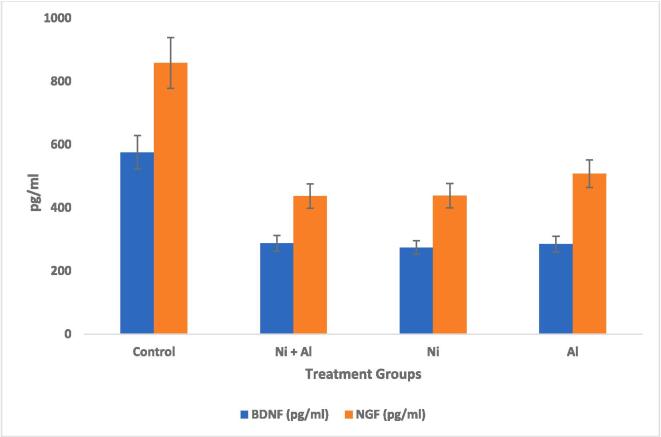
Effect of Ni, Al, and Ni & Al mixture on BDNF & NGF in frontal cortex in male albino rata.

Fig. 3, Fig. 4 show the effect of Ni, Al, and Ni & Al mixture on BDNF & NGF in the hippocampus and frontal cortex in male albino rats. The BDNF & NGF levels in the hippocampus and frontal cortex in male albino rats were significantly reduced following administration of Ni, Al, and Ni & Al mixture when compared with the control.

**Fig. 5:**
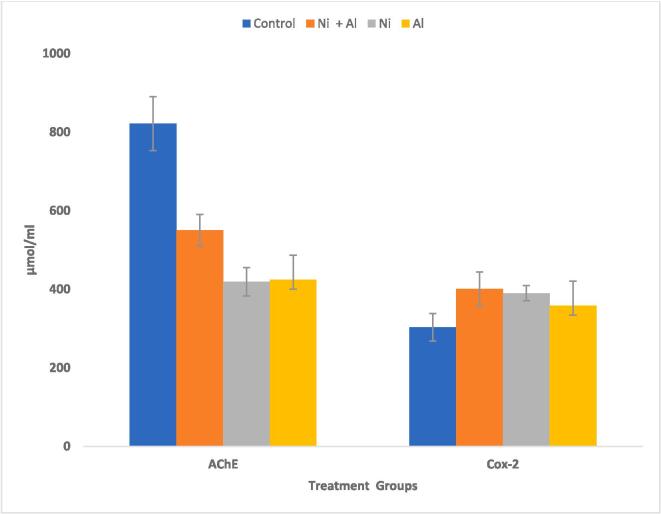
Effect of Ni, Al, and Ni & Al mixture on AChE & COX-2 on Hippocampus in male albino rats.

**Fig. 6:**
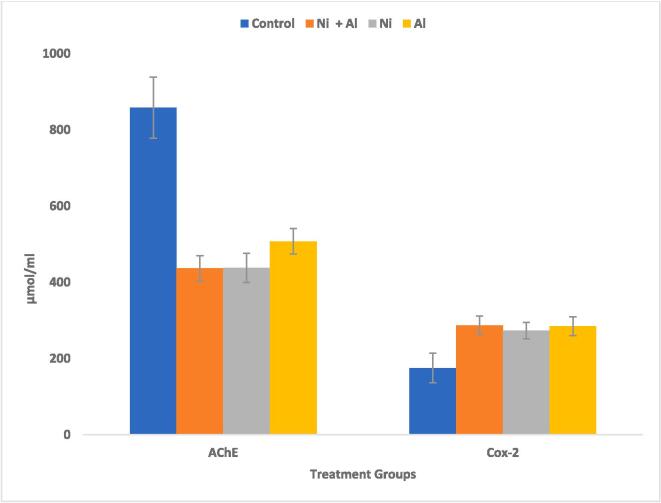
Effect of Ni, Al, and Ni & Al mixture on AChE & COX-2 on frontal cortex in male albino rats.

In comparison to the control, exposure to Ni, Al, and Ni & Al mixture caused significant (p > 0.05) decrease in AChE activity and significant (p > 0.05) increase in COX-2 activity in the hippocampus and frontal cortex of male rats Fig. 5, Fig. 6 respectively.

All in all, Ni/Al binary mixture exposed rats showed a shorter latency period of 3.21 **±** 1.40 s in comparison to 3.77 **±** 1.11(Ni only) and 3.99 **±** 1.16(Al only). Furthermore, Ni/Al binary mixture exposed rats had the lowest levels of Mg in both the hippocampus and frontal cortex when compared with the individual metals. In the hippocampus Al only, exposed rats significantly showed p < 0.05 higher iron and Ca levels in comparison to binary mixture. Whereas Al only exposed rats significantly showed p < 0.05 lower levels of iron but higher Ca than the Ni/Al binary mixture exposed rats. Exposure to Al only showed lower levels of BDNF in comparison to Ni/Al combination, whereas Ni/Al mixture had lower levels of NGF in comparison to the individual metals in the hippocampus. In the frontal cortex Ni only, exposed rats showed significantly lower levels of BDNF in comparison to Ni/Al mixture whereas the mixture showed significantly lower levels of NGF when compared with Al alone. There were higher levels of COX-2 in the Ni/Al mixture exposed rats than the individual metal treated rats in both the hippocampus and frontal cortex. In the same vein the AChE levels in the Ni/Al was higher than either Ni or Al alone in the hippocampus whereas in the frontal cortex. Ni/Al mixture exposed rats showed significantly lower AChE levels in comparison to Al only exposed rats.

## Discussion

The present study has evaluated a possible toxic role of the heavy metal mixture (HMM) comprising of Ni and Al, as well as the individual toxicity of Ni and Al only administration in a rat model. Understanding the toxicity of metals as mixtures is a true mimicry of real-life situations, given the emergence of public health maladies associated with exposure to a cocktail of metals. Environmental and occupational exposure to nickel at high levels can cause various adverse effects on human health, especially neurotoxicity (Lamtai et al., 2020). Some studies have linked Ni and Al mixture from food grade aluminum foil wrappers and other cooking utensils a (Dabonne et al., 2010, Duru and Duru, 2020). The ubiquity of metals in the environment is a public health concern. Human exposure through food or drinking water has led to extensive organ internalization, especially in the brain. Metals such as Al and Ni have been implicated in both the potentiation and induction of oxidative stress and inflammatory responses in tissue injury (Willhite et al., 2014). In this study there were no significant changes in body weight after 90 days of exposure to Ni, Al, and Ni/Al binary mixture in rats. There were however adipsia and aphagia on day 60 which might have contributed to the reduction in percent body weight gain within the period but were all reversed by day 90. This reversal might be a homoeostatic and adaptation mechanism to metalotoxicosis.

The higher level of Ni seen in the frontal cortex of rats exposed to Ni and Al mixture than Ni only exposed group may be suggestive of higher bioaccumulation and more toxicity than the individual Ni metal. This type of additive toxicity has been reported in metal mixture toxicity (Martin et al., 2021, Drakvik et al., 2020). Ni permeation into the brain occurs due to failures of the blood–brain barrier (BBB) or via the olfactory pathway (Gao et al., 2015, Lamtai et al., 2018, Xu et al., 2015), leading to eventual accumulation in the cerebral cortex and the whole brain (Gao et al., 2015, He et al., 2013, Lamtai et al., 2018) and triggering neurotoxicity (He et al., 2013) while disrupting neurotransmitters (David and Lobner, 2004, Jia et al., 2010). Al can disrupt the homeostasis of some metals, especially in the hippocampus and frontal cortex (Maya et al., 2016). Altered levels of essential metals are significantly associated with neurotoxicity induced by toxic metals (Bjørklund et al., 2020, Yu et al., 2020). The hippocampal and cortical levels of Al found in this study are comparable with a recent study that reported that Al exposure increases the content of Al in the hippocampus and midbrain and causes homeostasis of the content of other metals including and Fe in these tissues (Shang et al 2023). Fe, a redox-reactive metal, plays a critical role in sustaining normal brain function, as it is involved in the metabolic balance of nitric oxide (NO), neurotransmitter synthesis, and myelination, among others (Kim and Wessling-Resnick, 2014, Lane et al., 2018). In the present study, Ni/Al binary mixture exposed rats had the lowest levels of Mg in both the hippocampus and frontal cortex when compared with the individual metals. In the hippocampus, Al only exposed rats significantly showed p < 0.05 higher iron and Ca levels in comparison to binary mixture. Whereas Al only exposed rats significantly showed p < 0.05 lower levels of iron but higher Ca than the Ni/Al binary mixture exposed rats. Essential metals such as calcium (Ca), iron (Fe), and magnesium (Mg) play critical roles in maintaining normal function in the central nervous system (CNS) and participate in metabolic processes (Kawahara et al., 2018, Mezzaroba et al., 2019, Park et al., 2015)). Dyshomeostasis of these metals may interrupt normal physiological functions (Mezzaroba et al., 2019); for example, both deficiency and excessive accumulation of Fe can cause brain damage (Pasricha et al., 2021, Simunkova et al., 2019).

Most divalent heavy metals share similar chemical properties with the aforementioned metals and compete for metal-binding sites, transporters, and enzymatic proteins (Miller et al., 1990, Yu et al., 2020). Reduced brain magnesium levels can cause behavior and personality changes, apathy, irritability, and anxiety (Islam et al., 2013, Wacker and Parisi, 1968). Deficiency of calcium or magnesium may exacerbate anxiety, and a low Mg/Ca ratio may trigger the stress response (Seelig 1994). Mineral supplementation containing calcium and magnesium can significantly reduce anxiety, suggesting that alterations in essential trace elements may play a role in the pathogenesis of neurobehavioral deficits.

In this study, Al only exposure showed lower levels of BDNF in comparison to Ni/Al combination, whereas Ni/Al mixture had lower levels of NGF in comparison to the individual metals in the hippocampus. In the frontal cortex Ni only, exposed rats showed significantly lower levels of BDNF in comparison to Ni/Al mixture whereas the mixture showed significantly lower levels of NGF when compared with Al alone. Nerve growth factor (NGF) is a neurotrophin mainly found in the limbic system. It is recognized to be involved in cognition, mood, protection of neurons, neuroplasticity, and response to stress mechanisms (Shu and Mendell, 1999, Yeh et al., 2015). Brain-derived neurotrophic factor (BDNF) is the most abundant neurotrophin found in different compartments of the nervous system and is involved in neuronal development and differentiation (Blandini et al., 2006, Lipsky and Marini, 2007). NGF is a secreted growth factor that is important in the survival, growth, and maintenance of specific types of neurons in the central and peripheral nervous system. NGF is synthesized in the cortex and hippocampal cortical region of the brain and is required throughout maturity for cholinergic-hippocampal interaction (Allen et al., 2013). The brain-derived neurotrophic factor (BDNF) plays a crucial role in the survival and differentiation of neurons during development, and is highly expressed in the hippocampus and cortex, two brain regions important for learning and memory (Tao et al., 1998). A decrease in BDNF expression is associated with the development of several neurodegenerative diseases, and a reduction of BDNF following aluminum (Al) intoxication is consistent with previous studies (Ahmed et al., 2013, Ghoneim et al., 2015). Furthermore, Al can interfere with various enzymes involved in neurotransmitter biosynthesis (Abu-Taweel et al., 2012, Kawahara and Kato-Negishi, 2011).

Al can disrupt cellular and metabolic processes, including neurotransmission (Quintal-Tun et al., 2007), and experimental studies in rats have indicated that Al disrupts the cholinergic system, leading to neurobehavioral disturbances (Yellamma et al., 2010). Heavy metal-mediated cholinotoxicity has also been reported (Abdel-Salam et al., 2021, Abdel-Salam et al., 2016). In this study the AChE levels in the Ni/Al was higher than either Ni or Al alone in the hippocampus whereas in the frontal cortex. Ni/Al mixture exposed rats showed significantly lower AChE levels in comparison to Al only exposed rats. AChE plays a crucial role in cholinergic neurotransmission and is strongly associated with regulatory functions and neurobehavioral processes (Mesulam et al., 2002, Pari and Murugavel, 2007). The activity of AChE in rat brain synaptosomal plasma membranes following heavy metal exposure has been controversial, with some studies reporting an increase while others have reported a decrease (Goncalves et al., 2010, Moretto et al., 2004). AChE activity was enhanced in the cerebral cortex and hippocampus of rats exposed to 30 doses of subcutaneous mercury injections (0.1 mg/kg), while AChE inhibition was observed in different models of mercury intoxication (El-Demerdash, 2001, Goncalves et al., 2010). Exposure to both mercury and cadmium showed significant decreases in cortical AChE, while exposure to lead (Pb) resulted in high cerebrocortical ACh levels, suggesting that chronic exposure to low doses of heavy metals leads to a differential ACh response (Hrdina et al., 1976). Two plausible mechanisms of AChE inhibition by heavy metals include the displacement of metal cofactors from the active site or the direct deactivation of the enzyme site (Casalino et al., 1997, Goncalves et al., 2010). Once there is AChE inhibition, there is minimal synaptic hydrolysis of Ach and concomitant accumulation of Ach and cholinergic crisis (Goncalves et al., 2010, Olney et al., 1986). The diminution of AChE activity triggers cholinergic hyperactivity, confusion, headache, sleep disturbances, and memory lapses (Ecobichon, 2001, Goncalves et al., 2010). Since cerebral AChE activity is an important regulator of behavioral processes, the reduced AChE activity following heavy metal exposure may be an indicator of heavy metal-induced damage in the brain which has implication in the present study where Ni/Al mixture exposed rats showed significantly lower AchE levels in comparison to Al only exposed rats in this study.

Hippocampal brain-derived neurotrophic factor (BDNF) is essential for normal neuronal development and cognitive function (Gonzalez et al., 2016). The expression of BDNF is linked to neurotransmitter concentrations, and its synthesis is activated by neuronal activity and increased cytoplasmic Ca^2+^ levels via the activation of the transcription factor cyclic adenosine monophosphate (cAMP) responsive element binding protein (CREB) (Tao et al., 1998, Zheng et al., 2011). BDNF is neuroprotective via modulation of synaptic plasticity and function (Bathina and Das 2015) and is also reported to facilitate explicit memory encoding, storage, and retrieval of information in the hippocampal region of the brain (Bekinschtein et al., 2008). BDNF is a major contributor to energy homeostasis (Bothwell 1995), implying that increased BDNF levels may enhance cognitive capacity and potentially lead to reduced fatigue (Bathina and Das 2015). The mechanistic pathways of iron and BDNF interaction are not yet fully understood, but it is believed that optimal iron levels may be necessary for BDNF homeostasis (Texel et al., 2012). Hence, deficient brain Fe levels may downregulate BDNF with concomitant alterations in neurotransmitter levels (Texel et al., 2012), while excessive cerebral Fe levels may also reduce BDNF levels, resulting in cognitive and mental impairments (Radak et al., 2016, Sian-Hülsmann et al., 2011). This may arise from the meager and insufficient brain antioxidant defense machinery to handle noxious reactive oxygen species (ROS) provoked by high iron levels (Hwang, 2013, Radak et al., 2016, Sian-Hülsmann et al., 2011).

Under normal physiological conditions, ligands or signalling molecules activate signalling proteins inside the cell upon reaching receptor sites, which subsequently turn on the expression of specific target genes to produce proteins. However, in cases of heavy metal neurotoxicity, these metals reach the cell surface and compete with calcium ions for entry into the cell through the calcium channel. This competition ultimately deactivates the calcium-calmodulin pathway and prevents the signals from reaching the nucleus of the cell, leading to the deactivation of cAMP-response element binding protein (CREB). The activation of CREB protein is essential for the synthesis of cortical/hippocampal proteins such as BDNF (Jagasia et al., 2009). Therefore, heavy metal exposure directly modulates CREB activation, thereby impacting the synthesis of these proteins.

## Conclusion

In conclusion, this may be first or one of the few studies that evaluated the comparative toxicity of Ni, Al, and Ni/Al binary toxicity in small mammals. The present study confirms the susceptibility of the brain to the toxic effect of increased oral exposure to Ni and Al mixture in rats. The neurotoxicity of Al is likely to be the result of a combination of several mechanisms including oxidative brain injury and enhanced lipid peroxidation, disruption of neurotrophic, cholinergic functions, and induction of inflammation. Ni/Al mixture exposure provoked oxidative dysfunction in the hippocampus, frontal and cortex which were demonstrated by hippocampal and cortical bioaccumulation of Al and Ni, decrease in antioxidant parameters (CAT, SOD, GSH, GPx) and elevation of marker lipid peroxidation – MDA. There was also upregulation of COX-2 and down regulation of both BDNF, NGF and AChE which adversely affected behaviour in Barnes performance test. The BDNF-COX-2 AchE signalling pathway may be involved in the neurotoxicity of binary mixture of Ni and Al in male rats. Taken together exposure to Ni, Al, and Ni & Al mixture showed significantly (p < 0.05) longer time in locating the escape hole indicative of impairment in learning and spatial memory in comparison to the control which correlated with the significantly lower levels of BDNF & NGF in these Ni, Al, and Ni & Al mixture exposed groups.

## CRediT authorship contribution statement

**Chidinma P. Anyachor:** . **Chinna N. Orish:** Conceptualization. **Anthonet N. Ezejiofor:** Supervision. **Ana Cirovic:** . **Aleksandar Cirovic:** . **Kenneth M. Ezealisiji:** . **Orish E. Orisakwe:** Conceptualization.

## Declaration of Competing Interest

The authors declare that they have no known competing financial interests or personal relationships that could have appeared to influence the work reported in this paper.

## Data Availability

No data was used for the research described in the article.
